# Genomic Surveillance of *Plasmodium falciparum* Drug Resistance Markers Between October 2021 and June 2023 in Kigali, Rwanda

**DOI:** 10.3390/pathogens14111092

**Published:** 2025-10-27

**Authors:** Sandra Noukimi Fankem, Jean-Bosco Mbonimpa, Edgar Mutebwa Kalimba, Mariama Telly Diallo, Mary Efeti Teke, Jacob Souopgui

**Affiliations:** 1 Laboratory of Embryology and Biotechnology, Department of Molecular Biology, Faculty of Science, Université Libre de Bruxelles, 6041 Gosselies, Belgium; sandra.fankem.noukimi@ulb.be (S.N.F.); edgar.kalimba@ulb.bemariama.telly.diallo@ulb.be (M.T.D.); 2 Rwanda Malaria Research Laboratory, King Faisal Hospital Rwanda, Kigali P.O. Box 2534, Rwanda; furerebosco@gmail.com; 3 Department of Gerontology, Faculty of Medicine and Pharmacy, Vrije Universiteit Brussel, 1090 Brussels, Belgium; mary.teke.efeti@vub.be; 4 Department of Biochemistry and Molecular Biology, Faculty of Science, University of Buea, Buea P.O. Box 63, Cameroon

**Keywords:** drug resistance, genomic surveillance, kelch13, SNPs

## Abstract

Artemisinin-based combination therapies (ACTs) remain the cornerstone of malaria treatment in Rwanda, but the emergence of drug resistance threatens their efficacy. This study conducted genomic surveillance of *Plasmodium falciparum* isolates collected in Kigali between October 2021 and June 2023 to assess resistance markers. Using Oxford Nanopore Technology and Sanger sequencing methods, we analyzed 250 clinical isolates focusing on mutations in the *pfcrt*, *pfmdr1*, *pfdhfr*, *pfdhps*, and *Pfkelch13* genes. Resistance-associated mutations were highly prevalent: *pfcrt* 76T (26%) and *pfmdr1* 184F (72.8%) were common, indicating continued lumefantrine pressure. All isolates carried mutations in *pfdhfr* and *pfdhps*, with the **IRN**I-SA**E**AA and **IRN**I-SA**EG**A haplotypes found in 45.6% and 24.8% of samples, respectively, suggesting sustained antifolate resistance. *Pfkelch13* mutations were present in 50.4% of isolates, including validated R561H (25.6%), A675V and candidate P441L mutations. Novel haplotypes, including K189T + R561H (24.8%), were identified for the first time in Rwanda. The BTB/POZ domain mutation H384R was observed in 6.4% of isolates, raising questions about its potential functional role. These findings highlight complex and evolving resistance patterns and emphasize the urgent need for continued molecular surveillance and functional validation to inform malaria control strategies in Rwanda.

## 1. Introduction

Malaria continues to be a leading public health challenge worldwide, with an estimated 263 million cases and 597,000 deaths reported in 2023, primarily in sub-Saharan Africa [1]. *Plasmodium falciparum* is the most lethal of the human malaria parasites and accounts for over 95% of malaria cases in Africa [1]. In Rwanda, malaria transmission is unstable and seasonal, with recent years showing fluctuations in incidence due to a combination of control measures, climatic changes, and emerging drug resistance. Although Rwanda experienced a decline in malaria cases from 2018 to 2022 following intensified control strategies, a resurgence has been observed, necessitating close monitoring of parasite evolution and drug susceptibility [2,3,4].

The emergence and spread of antimalarial drug resistance is a major threat to global malaria control and elimination efforts. Historically, widespread resistance to chloroquine (CQ) and sulfadoxine-pyrimethamine (SP) led to changes in national treatment policies across Africa [5,6]. Rwanda replaced CQ with amodiaquine-SP in 2001, followed by a shift to artemether-lumefantrine (AL) as the first-line therapy in 2006 [7,8]. However, resistance markers linked to previously used drugs remain prevalent in the parasite population [9,10], likely sustained by selective pressures from related medications and cross-border transmission. The threat of antimalarial drug resistance compromises both treatment and prevention strategies. Genomic surveillance of key *P. falciparum* resistance markers, including *pfcrt*, *pfmdr1*, *pfdhfr*, *pfdhps*, and *kelch13*, is essential for guiding therapeutic policies.

The *pfcrt* gene encodes a transporter protein associated with CQ resistance; the K76T mutation is particularly critical [11,12]. Although CQ is no longer used, the wild-type K76 allele has not fully re-established in Rwanda, suggesting residual fitness advantages of resistant strains or ongoing selection pressures. The *pfmdr1* gene influences resistance to multiple drugs, including lumefantrine, mefloquine, and amodiaquine. Variants such as N86Y and Y184F modulate drug response and are monitored for changes in AL efficacy [12]. Resistance to SP is largely conferred by point mutations in the *pfdhfr* and *pfdhps* genes. A combination of triple *dhfr* mutations (N51I, C59R, S108N) and double *dhps* mutations (A437G, K540E) forms the quintuple mutant, which is strongly associated with SP treatment failure [13]. Despite discontinuation of SP for treatment, the high prevalence of these mutations persists in Rwanda [14,15].

The *kelch13* gene has emerged as a molecular marker for artemisinin resistance, with several non-synonymous mutations in the propeller domain associated with delayed parasite clearance. Of particular concern is the R561H mutation, which has been identified in Rwanda and shown to be expanding clonally, marking one of the first confirmed cases of indigenous artemisinin resistance in Africa [16,17]. Evidence from therapeutic efficacy studies links the R561H mutation to prolonged parasite clearance times and reduced efficacy of ACTs, raising alarms for treatment outcomes and prompting the need for intensified surveillance and response measures [18]. Resistance to artemisinin in *Plasmodium falciparum* has been increasingly linked not only to mutations in the well-known *kelch13* propeller domain but also to mutations in the BTB/POZ domain of the *kelch13* gene. Paloque et al. (2022) demonstrated that mutations within the BTB domain can modulate artemisinin susceptibility, potentially affecting the parasite’s stress response pathways and contributing to delayed parasite clearance in clinical infections [19]. These findings suggest a broader functional role of the *kelch13* protein domains in mediating artemisinin resistance beyond the propeller region alone, underscoring the need for comprehensive genomic surveillance of multiple *kelch13* regions to better predict and manage resistance emergence.

Given the increasing reports of resistance-associated mutations in Kigali and across Rwanda, ongoing genomic surveillance is essential to detect and respond to evolving *P. falciparum* resistance patterns. Monitoring mutations in *pfcrt*, *pfmdr1*, *pfdhfr*, *pfdhps*, and *kelch13* can provide early warning signals for declining drug efficacy and guide strategic decisions in malaria control and elimination programs.

## 2. Materials and Methods

### 2.1. Sample Collection and DNA Extraction

This study was designed as a repeated cross-sectional analysis using residual blood samples collected from patients attending King Faisal Hospital in Rwanda between October 2021 and June 2023. Kigali is situated at approximately 1500 m above sea level and experiences a temperate tropical climate with two rainy seasons: March–May and October–December. Malaria transmission in Kigali is generally low to moderate, with *Plasmodium falciparum* being the predominant species. The city is characterized by urban and peri-urban settings, with variable access to healthcare facilities and vector control measures.

A total of 310 samples were included, each representing a unique patient encounter during the study period. These were leftover blood samples originally obtained for routine malaria diagnosis. The residual blood was subsequently collected and preserved by mixing with DNA/RNA Shield (Zymo Research, Tustin, CA, USA), following the manufacturer’s instructions. Samples were stored at −20 °C and later transported via cold chain to the Embryology and Biotechnology Laboratory, ULB in Belgium for further molecular analysis. Total genomic DNA was extracted from blood collected during the study using Maxwell^®^ RSC Whole Blood DNA Kit (Promega—AS1520, Madison, WI, USA). A total 300 µL of diluted blood samples were used for DNA extraction and a volume of 50 µL was used for the elution.

### 2.2. Amplicon Generation and Sequencing

A total of four *Plasmodium falciparum* genes, namely *crt*, *mdr1*, *dhfr*, and *dhps,* were amplified using a multiplex PCR approach. Primer sequences and PCR conditions were applied as previously described [20]. In addition, the *kelch13* gene was amplified separately using conventional PCR with primers designed using Primer3: forward primer Kelch13-F (5′-GATGCAGCAAAT CTT ATA AATGATGATTCT-3′) and reverse primer Kelch13-R (5′-GCCAAGCTGCCATTCATTTG-3′). Each 25 µL PCR reaction for *kelch13* consisted of 5 µL of genomic DNA, 12.5 µL of 2X GoTaq^®^ Hot Start Green Master Mix (Promega, Madison, WI, USA), and 2 µL each of forward and reverse primers (10 µM). Amplification conditions included an initial denaturation at 95 °C for 5 min, followed by 39 cycles of 95 °C for 15 s, 60 °C for 30 s, and 72 °C for 1 min, with a final extension at 72 °C for 3 min. Molecular-grade water was used as a negative control.

PCR products were pooled and visualized on a 1.5% agarose gel alongside a 1 kb DNA ladder. PCR products were purified using Wizard^®^ SV Gel and PCR Clean-Up System (Promega—A9281, Madison, WI, USA) according to manufacturer’s instruction. Library preparation was performed using Ligation sequencing amplicons—Native Barcoding Kit 24 V14 (SQK-NBD114.24, Oxford, UK) from Oxford Nanopore Technology (ONT, Oxford, UK). Prepared library was run on Flongle Flow Cells (R10.4.1) for 24 h using MinKNOW software version 24.06.16 (ONT, Oxford, UK). Of note, the throughput of Flongle flow cells is lower and per-sample yields can vary due to barcode efficiency. Therefore, sequencing was run for extended time to ensure that all samples reached optimal coverage.

### 2.3. Bioinformatics Processing and Analysis

Basecalling and barcoding were performed using Guppy v7.0.9 integrated within MinKNOW v23.07.5. Basecalling and demultiplexing parameters were configured with a minimum barcode quality score of 60 and a minimum read quality score of 9. No filter was applied for minimum read length. Reads generated were mapped on *P. falciparum* 3D7 reference genome using Minimap2 software version 2.28-r1209 [21], generating a SAM file. BAM statistics, filtration, sorting, and indexing were performed using different Samtools version 1.21 [22] functionalities (flagstat, view, sort, and index) to obtain sorted Bam and their indexes. BAM quality control was performed using the Qualimap software version 2.2.2 [23] and BAM files were visualized using IGV software version 2.16.0 [24]. Variance calling was performed using Clair3 Clair3 version 1.0.10 [25] haploid calling by including –include_all_ctgs, –haploid_sensitive and –gvcf parameters. The SNPs in gVCFs were filtered by quality (>10) and depth (>20) using bcftools version 1.21 [22] and annotation to extract SNPs information was performed using snpEff version 4.10 [26]. All data generated were analyzed and plotted using GraphPad Prism version 8.

### 2.4. Sequencing Validation Using Sanger Method

A total of 90 samples were randomly selected and amplified using nested PCR targeting *kelch13* gene. Briefly, *kelch 13* amplification and purification was performed as described above. Purified products were then diluted and mixed with forward primer and sent for sequencing to Eurofins genomics as described by manufacturer. Fasta sequences generated were aligned using UGene software version 45.0.

## 3. Results

A total of 310 blood samples were collected between October 2021 and June 2023. Participants were predominantly male (60.3%), and most were aged 21–50 years (48.1%). Other age groups included 6–20 years (23.9%), 0–5 years (15.8%), and over 50 years (12.3%) as shown in Table 1 below. Parasitaemia levels were mostly low to moderate, with 41.0% of individuals showing <1000 parasites/µL, 41.6% between 1000 and 10,000 parasites/µL, and 17.4% exceeding 10,000 parasites/µL.

**Figure 1:**
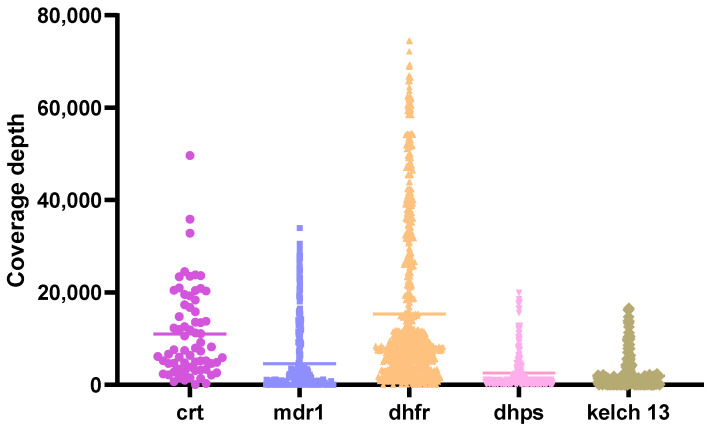
Coverage depth of targeted genes. Multiplex PCR was performed to amplify *crt*, *mdr1*, *dhfr* and *dhps* genes, while *kelch13* was amplified separately. The resulting PCR products were pooled and visualized on a 1.5% agarose gel electrophoresis. Purified amplicons were then used for library preparation using the Oxford Nanopore Technologies (ONT) Native Barcoding Kit 24 V14 (SQK-NBD114.24). The final library was loaded onto a Flongle flow cell (R10.4) and sequenced for over 20 h. Raw FASTQ files generated from the run were processed to produce variant call format (VCF) files. Variant coverage depths were evaluated, with a minimum threshold set at 20×.

Of the 310 samples, 250 (80.6%) were successfully amplified by PCR for all five targeted *Plasmodium falciparum* genes. The unsuccessful amplifications were attributed to low or negative parasitemia by microscopy leading to PCR amplification failure. The 250 samples that showed successful amplification of all target genes were included in downstream analyses, including variant calling and haplotype analysis. All drug-resistant genes were analyzed to identify variants supported by a minimum coverage depth of >20 reads (Figure 1).

Single nucleotide polymorphisms (SNPs) were detected across all targeted loci, with key mutations observed at known resistance-associated positions in *crt*, *mdr1*, *dhfr*, *dhps*, and *kelch13*.

### 3.1. Molecular Markers Associated with Aminoquinolines Drug Resistance

**Figure 2:**
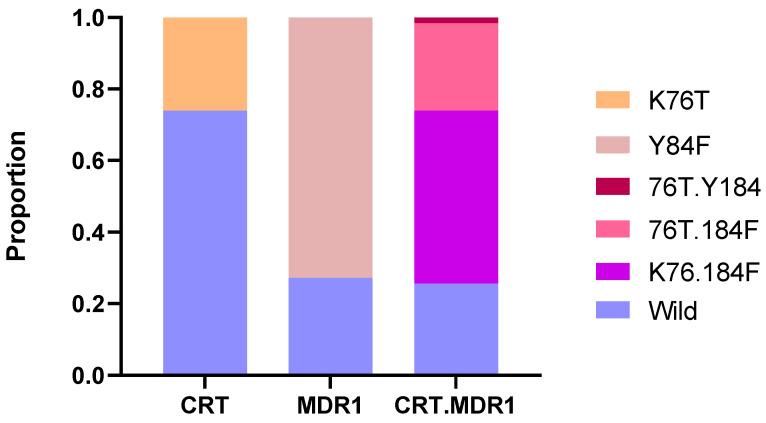
Distribution of *Plasmodium falciparum* SNPs in CRT and MDR1 across the sequenced samples. The stacked bar chart shows the proportion of wild-type and mutant alleles for the CRT and MDR1 genes individually, as well as for combined CRT + MDR1 haplotypes.

Drug resistance markers were evaluated by amplifying and sequencing key genetic markers associated with antimalarial resistance, namely *crt*, *mdr1*, *dhfr*, *dhps* and *kelch13*. VCF files obtained after processing the ONT data were analyzed and revealed in 74.4% (186/250) the presence of at least one resistance marker on *pfcrt* and *pfmdr1* genes (Figure 2). The *pfcrt* K76 wild type was observed in 74% (185/250) of the samples while the mutant 76T was identified in 26% (65/250). The wild-type Y184 allele of the *pfmdr1* gene was observed in 27.2% (68/250) of the samples while the mutant 184F was predominant and found in 72.8% (182/250). For *crt* and *mdr1* haplotype, the K76.184F haplotype was predominant with 48.4% (121/250).

### 3.2. Molecular Markers Associated with Antifolate Drug Resistance

**Figure 3:**
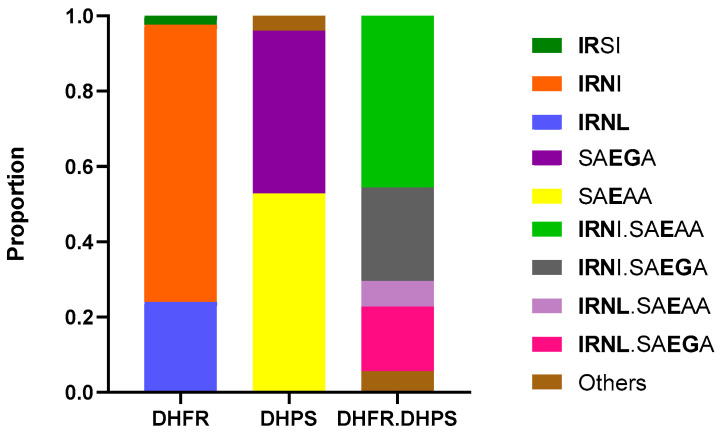
Distribution of *Plasmodium falciparum* haplotypes in DHFR, DHPS in Kigali, Rwanda. DHFR haplotypes correspond to amino acid positions 51, 59, 108, and 164 (wild type, NCSI) and DHPS haplotypes correspond to amino acid positions 436, 437, 540, 581, and 613 (wild type, SAKAA). DHFR-DHPS haplotypes represent the combination of *dhfr* and *dhps* gene variants, and mutations in both genes provide insights into the efficacy of sulphadoxine-pyrimethamine treatment.

Regarding antifolates drug resistance markers (Figure 3), all isolated carried at least one SNP in either the *pfdhfr* or *pfdhps* genes. Haplotype analysis of the DHFR genotypes showed that the triple mutant **IRN**I haplotype was the most predominant combination (73.6%, 184/250), followed by the quadruple mutant **IRNL** (24%, 60/250) and double mutant **IR**SI (2.4%, 6/250) which refers to amino acid positions 51, 59, 108 and 164 (with 108N, the main allele to pyrimethamine resistance). Two main haplotypes were identified in DHPS with predominantly the single mutant (K540E) SA**E**AA (52.8%, 132/250) followed by the double mutant SA**EG**A (43.2%, 108/250) to amino acid positions 436, 437, 540, 581 and 613. Overall, the DHFR and DHPS haplotype in our study displayed many combinations with the quadruple **IRN**I.SA**E**AA (45.6%, 114/250), quintuple IRNI.SAEGA (24.8%, 62/250) and sextuple mutants **IRNL**.SA**EG**A (17.2%, 43/250) being the most prevalent.

### 3.3. Identification of SNPs in the kelch13 Gene

Artemisinin molecular markers of resistance were assessed by sequencing across the N-terminal/CCC, BTB/POZ, and propeller domains of the *Pfkelch13* gene. Among 250 *Plasmodium falciparum* isolates, 50.4% (124/250) carried at least one mutation in the *kelch13* gene. As described in Table 2, the most prevalent mutation was K189T (31.2%; 78/250), located in the N-terminal region, although its role in artemisinin resistance remains unknown. Notably, the R561H mutation, validated by the WHO as a molecular marker of partial artemisinin resistance [18], was present in 25.6% of isolates (64/250). Other propeller domain mutations included P441L (2.0%) and P574L (0.4%), both considered candidate markers of resistance, and A675V (2.0%), which is also validated. Rare mutations such as C469F, A569V, A578S, P667R, and F699C were each observed in 0.4–2.8% of isolates, though their roles in drug resistance are currently unclear.

In the BTB/POZ domain, the H384R mutation was found in 6.4% of isolates, and while not yet validated, recent clinical findings from a longitudinal study in Rwanda suggest that this influences artemisinin susceptibility by altering protein structure and interaction with the Cullin 3 Ligase enzyme complex.

**Figure 4:**
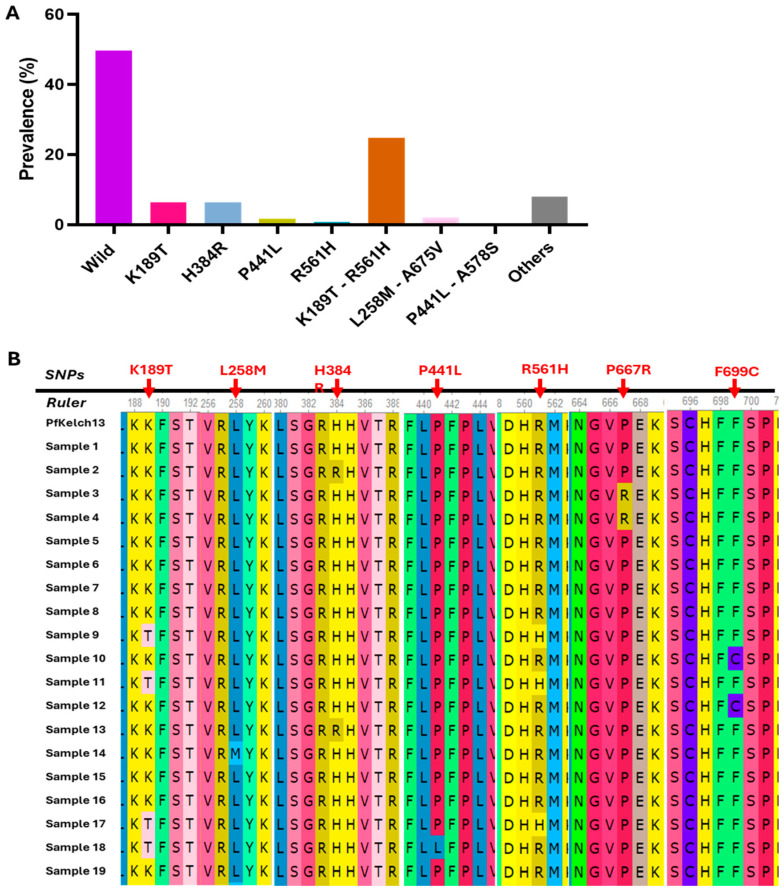
Overall SNP and haplotypes identified in the *kelch13* gene. (**A**) Histogram presenting the proportions of *Kelch13* SNP and haplotypes. (**B**) Multiple sequence alignment of 19 *kelch13* sequences generated from Sanger sequencing data.

Looking at the overall mutations (Figure 4A), *kelch13* haplotypes were observed in our study. Principally, the combination K189T + R561H was predominant and appeared in 24.8% of isolates (62/250) as compared to the single validated mutation R561H that was present in 0.8% (2/250) and a non-validated K189T mutation that was present in 6.4% (16/250). Also, less frequent combinations included L258M + A675V (2.0%, 5/250) and P441L + A578S (0.4%, 1/250).

A subset of 90 samples was randomly selected from the full collected clinical isolates and subjected to Sanger sequencing to validate the *kelch13* mutations initially identified ONT sequencing. This orthogonal sequencing approach was used to ensure the accuracy and reliability of mutation calls, particularly for key resistance-associated variants. A representative multiple sequence alignment of 19 samples is presented in Figure 4B, illustrating the consistency of observed mutations between Sanger and ONT data. This alignment confirms the presence of several mutations, including the validated R561H and A675V as well as candidate or uncharacterized variants such as P441L, and F699C, and the haplotypes K189T + R561H, reinforcing the robustness of ONT-based mutation detection in this study.

## 4. Discussion

This study provides an updated overview of *Plasmodium falciparum* drug resistance in Kigali, Rwanda, through genomic surveillance conducted between October 2021 and June 2023. By targeting key molecular markers associated with antimalarial resistance, we aimed to assess the current prevalence of resistance-linked mutations and their potential implications for treatment efficacy in Rwanda. Hence, of the 310 samples, 250 (80.6%) were successfully amplified by PCR for all five targeted *Plasmodium falciparum* genes. The unsuccessful amplifications were attributed to low or negative parasitemia by microscopy leading to PCR amplification failure, suggesting that some patients were pre-exposed to malaria drugs or presenting with malaria-like symptoms as well known for infectious disease [27]. It is a common practice in malaria-endemic countries for patients to access and take drugs before consulting a health facility when the symptoms persist [28].

The persistence of mutations in *pfcrt* and *pfmdr1* was observed and reflects the ongoing selective pressure imposed by historical and current antimalarial drug use in Rwanda. Despite the national shift from CQ to ACT in the mid-2000s, the *pfcrt* K76T mutation, associated with CQ resistance remained present in 26% of isolates. Although a partial reversion to the wild-type K76 allele was seen in 74%, this suggests incomplete recovery of CQ susceptibility, consistent with regional reports of persistent 76T alleles years after CQ withdrawal [12,29,30]. The high prevalence of the *pfmdr1* 184F mutation (72.8%) and reduced presence of the wild-type Y184 (27.2%) further supports the idea that drug pressure from lumefantrine (partner drug in AL) continues to shape *Plasmodium falciparum* genetic profiles [12]. These findings are aligned with previous studies in Rwanda and East Africa, where *pfmdr1* 184F has been associated with reduced susceptibility to lumefantrine and potential selection under ACT use [31,32,33]. The presence of both *pfcrt* 76T and *pfmdr1* 184F mutations in most isolates (74.4%) combined with the WHO-validated and emerging SNPs in K13 suggest a reduced efficacy of AL in some subpopulations as reported recently in Uganda [34]. These findings warrant continued monitoring of treatment outcomes and the genetic landscape of drug resistance in Rwanda.

Our findings also demonstrated a high prevalence of antifolate resistance markers in *Plasmodium falciparum* isolates collected in Kigali. All samples carried at least one SNP in either the *pfdhfr* or *pfdhps* gene, with the triple mutant **IRN**I haplotype (N51I, C59R, S108N) observed in 73.6% of isolates. This was followed by the quadruple **IRNL** (24.0%) and double mutant **IR**SI (2.4%). These mutations, particularly S108N, have long been associated with high-level resistance to pyrimethamine and are widespread across sub-Saharan Africa [14,29]. On the *pfdhps* gene, the single mutant SA**E**AA (K540E) and the double mutant SA**EG**A (A437G and K540E) were found in 52.8% and 43.2% of isolates, respectively. These mutations are known to confer resistance to sulfadoxine, and their high frequency is consistent with previous studies in Rwanda as well as regional data from East Africa [35,36,37]. The combined analysis of *pfdhfr-pfdhps* haplotypes revealed that quadruple (**IRN**I-SA**E**AA), quintuple (**IRN**I-SA**EG**A), and sextuple (**IRNL**-SA**EG**A) mutant combinations were present in 45.6%, 24.8%, and 17.2% of isolates, respectively. These genotypes have been strongly associated with SP treatment failure and reduced effectiveness of intermittent preventive treatment in pregnancy (IPTp) [14,38].

Regarding genotyping of *pfkelch13* gene, our study revealed a concerning level of molecular diversity, with 50.4% of *Plasmodium falciparum* isolates from Kigali carrying at least one non-synonymous mutation. While the most common variant detected was K189T (31.2%), located in the N-terminal domain, its functional role in artemisinin resistance remains unclear, consistent with prior findings in Rwanda and Uganda where its prevalence has also been noted but not associated with delayed parasite clearance [17,39]. However, the validated resistance marker R561H was detected in 25.6% of isolates, supporting its continued spread in Rwanda from 7.5% in 2015 [18]. This study also revealed the presence of *Pfkelch13* haplotypes for the first time. The most frequent was K189T + R561H, detected in 24.8% of samples, exceeding the frequency of isolates harbouring R561H alone (0.8%) or K189T alone (6.4%). This observation raises the hypothesis of potential epistatic interactions between domains, which may enhance the fitness or transmissibility of resistant strains. The detection of additional haplotypes such as L258M + A675V and P441L + A578S, although at lower prevalence, further highlights the increasing complexity of *Pfkelch13* evolution under drug pressure. The detection of other propeller domain mutations such as P441L, P574L, and A675V, including two WHO-categorized candidate and one validated marker, respectively, further suggests sustained selective pressure from artemisinin-based therapies.

Kigali, a growing cosmopolitan city, is characterized by high population mobility, including immigrants and people travelling for international events, business, and tourism. This mobility could lead to imported *Plasmodium falciparum* strains, contributing to the expansion of the local parasite subpopulation. The current study did not address this hypothesis, but investigating the role of parasite importation, particularly in the context of drug resistance emergence and spread, will be a key priority in our future research.

Finally, we observed mutations in the BTB/POZ domain, specifically H384R in 6.4% of isolates, a relatively high frequency. Though not currently classified as a validated or candidate marker, emerging structural analyses suggest that mutations in the BTB/POZ domain can alter the substrate recognition and degradation properties of the Kelch13 protein, potentially contributing to artemisinin resistance [19]. This underlines the need for further functional studies into the role of non-propeller domain mutations.

## 5. Conclusions

This study highlights the evolving landscape of *Plasmodium falciparum* drug resistance in Kigali, a growing cosmopolitan city of Rwanda, emphasizing the continued evolution and persistence of resistance biomarkers. The observed mutation patterns suggest ongoing drug pressure and the potential for reduced efficacy of current antimalarial therapies. Our findings underscore the necessity of sustained genomic surveillance, integration with therapeutic efficacy data, and targeted functional studies to inform national treatment policies and guide effective containment strategies.

## Figures and Tables

**Table 1:** Baseline characteristics of study participants.

Characteristics		Frequency N (%)
Gender	Male	187 (60.32)
	Female	123 (39.68)
Age (0–78 years)	0–5	49 (15.81)
	6–20	74 (23.87)
	21–50	149 (48.06)
	>50	38 (12.26)
Parasitemia (parasites/µL of blood)	<1.000	127 (40.97)
	1.000–10.000	129 (41.61)
	>10.000	54 (17.42)

**Table 2:** Prevalence and status of *Pfkelch13* mutations detected in *Plasmodium falciparum* isolates from Kigali.

Gene	Target Region	Mutation	Status	Prevalence % (n)
*Kelch13*	N-terminal and CCC	K189T	Unknown	31.2% (78/250)
		T207K	Emerging	0.4% (1/250)
		R255K	Emerging	0.4% (1/250)
		L258M	Unknown	2.4% (6/250)
	BTB/POZ	H384R	Emerging	6.4% (16/250)
	Propeller	P441L	Candidate	2.0% (5/250)
		C469F	Emerging	0.4% (1/250)
		R561H	Validated	25.6% (64/250)
		A569V	Emerging	1.2% (3/250)
		P574L	Candidate	0.4% (1/250)
		A578S	Emerging	0.4% (1/250)
		P667R	Emerging	2.0% (5/250)
		A675V	Validated	2.0% (5/250)
		F699C	Emerging	2.8% (7/250)

## Data Availability

The genome sequence data presented in this study are available from the European Nucleotide Archives (ENA) database under the accession number: PRJEB101030 (ERP182457).

## Abbreviations

The following abbreviations are used in this manuscript:

ACT	Artemisinin-based combination therapy
AL	Artemeter–Lumefantrine
BTB/POZ	Broad-complex, Tramtrack, and Bric-à-brac/POxvirus and Zinc finger
CCC	Coiled-coil containing
CQ	Chloroquine
CRT	Chloroquine resistance transporter
DHFR	Dihydrofolate Reductase
DHPS	Dihydropteroate Synthase
MDR1	Multidrug resistance 1
ONT	Oxford Nanopore technology
PCR	Polymerase chain reaction
SNP	Single nucleotide polymorphism
SP	Sulfadoxine-pyrimethamine
